# Supplementary figures and images for: Co-targeting B-RAF and PTEN Enables Sensory Axons to Regenerate Across and Beyond the Spinal Cord Injury
Source: Front Mol Neurosci. 2022 Apr 26;15:891463. doi: 10.3389/fnmol.2022.891463 (PMC9087900; doi:10.3389/fnmol.2022.891463)

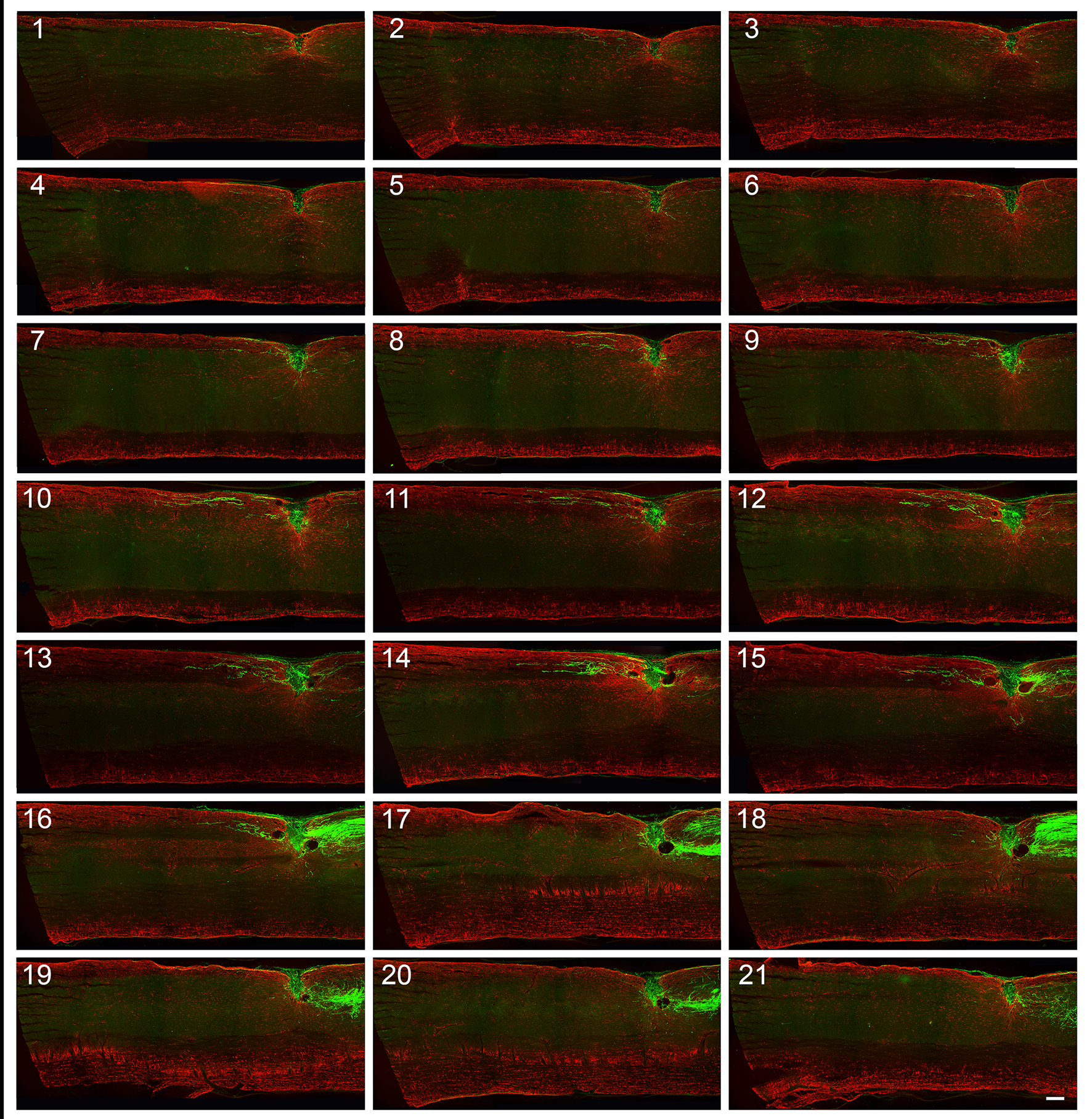

Supplement: Supplementary Figure 1 — Serial sections from the kaBRAF/PTEN iTg mouse spinal cord shown in Figure 1D. Numerous regenerating sensory axons penetrate the lesion epicenter and extend long distances rostral to the lesion site at 3 weeks after SCI. DC axons are labeled by injecting scAAV2-eGFP into right L4 and L5 DRGs. #16 image is also shown in Figure 1D. Scale bar: 200 μm. [file Image_1.TIF]

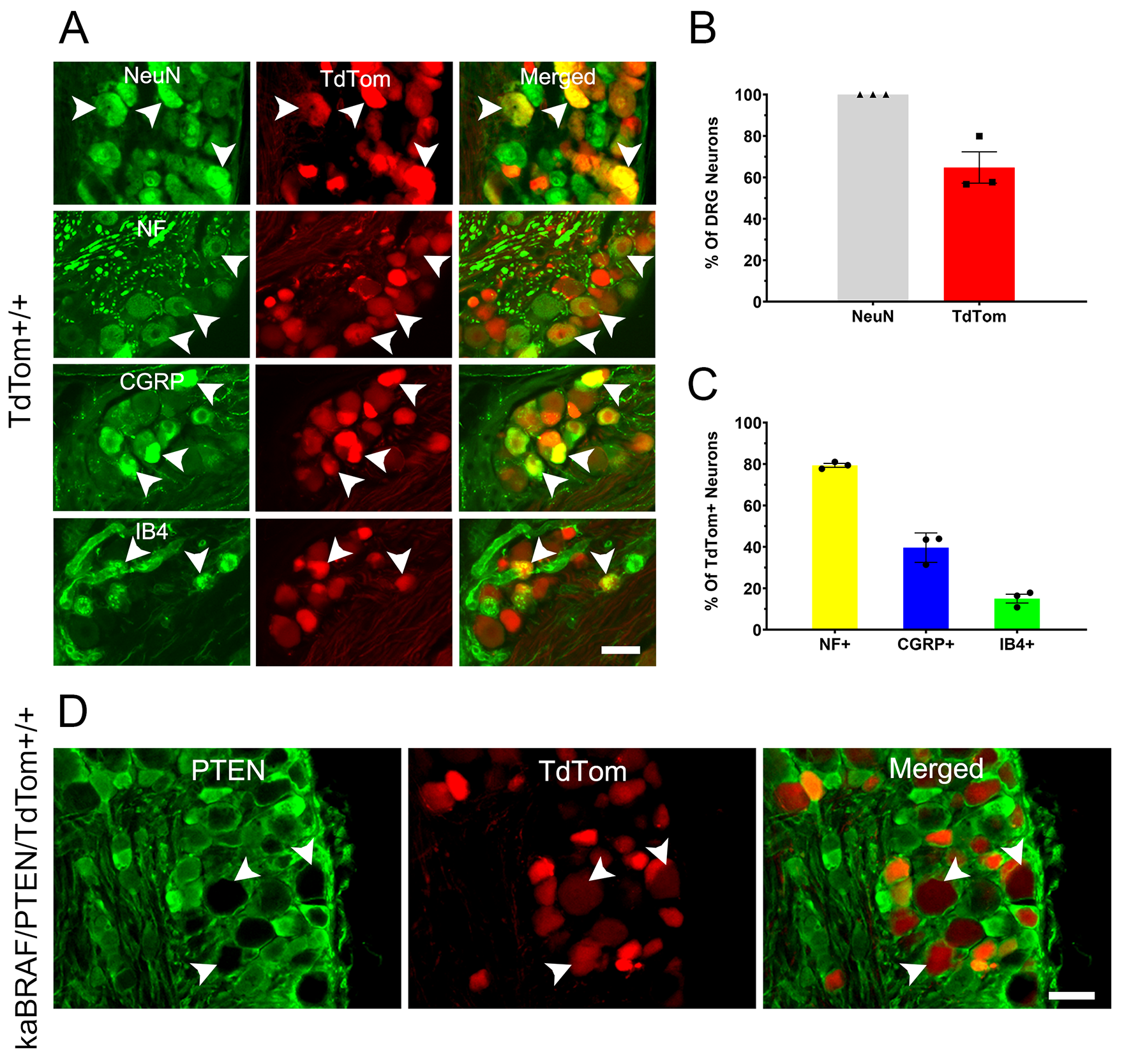

Supplement: Supplementary Figure 2 — Viral and genetic targeting of B-RAF and PTEN. (A) Representative image of a R26-TdT mouse DRG microinjected with scAAV2-Cre and co-stained with the global mature neuronal marker NeuN, large-diameter neuron marker NF, small-diameter peptidergic neuron marker CGRP, and small diameter non-peptidergic neuron marker IB4. (B) Quantitative analysis of transfected [NeuN+/TdTom+, arrowheads in panel (A)] mature neurons as a percentage of total (NeuN+) DRG neurons. Single unilateral microinjection of scAAV2-Cre into R26-TdT mice transfected > 63.6% of mature neurons in the DRG. (C) Quantitative analysis of transfected large diameter (NF+/TdTom+), peptidergic (CGRP+/TdTom+), and non-peptidergic [IB4+/TdTom+, arrowheads in panel (A)] DRG neurons. Single unilateral microinjection of a scAAV2-Cre into R26-TdT mice transfected > 79.4% of NF+ large-diameter neurons, 39.6% of small-diameter peptidergic CGRP+ neurons, and 15% of small-diameter non-peptidergic IB4+ neurons in the DRG. (D) Representative image of an LSL-kaBRAF:PTENf/f:R26-TdT mouse DRG microinjected with scAAV2-Cre and co-stained with the PTEN antibody showing high TdTom expression and PTEN deletion (arrowheads) in sensory neurons upon Cre-mediated recombination. PTEN deletion is particularly evident in large-diameter neurons. Scale bar, 50 μm (A). [file Image_2.TIF]
